# Factors Associated with Increased Risk of Urosepsis during Pregnancy and Treatment Outcomes, in a Urology Clinic

**DOI:** 10.3390/medicina59111972

**Published:** 2023-11-08

**Authors:** Viorel Dragos Radu, Radu Cristian Costache, Pavel Onofrei, Liviu Antohi, Razvan Lucian Bobeica, Iacov Linga, Ingrid Tanase-Vasilache, Anca Irina Ristescu, Alina-Mariela Murgu, Ionela-Larisa Miftode, Bogdan Alexandru Stoica

**Affiliations:** 1 Department of Urology, Faculty of Medicine, University of Medicine and Pharmacy “Gr. T. Popa”, 700115 Iasi, Romania; viorel.radu@umfiasi.ro (V.D.R.); radu.costache@umfiasi.ro (R.C.C.); 2 Urological Department, “C.I. Parhon” University Hospital, 700115 Iasi, Romania; bobeica.razvan-lucian@d.umfiasi.ro (R.L.B.); iacov_linga@email.umfiasi.ro (I.L.); 3 Department of Morpho-Functional Sciences II, Faculty of Medicine, University of Medicine and Pharmacy “Gr. T. Popa”, 700115 Iasi, Romania; 4 Urological Department, Elytis Hope Hospital, 700010 Iasi, Romania; 5 Department of Anaesthesia and Intensive Care, “C.I. Parhon” University Hospital, 700115 Iasi, Romania; liviuantohi70@gmail.com; 6 Department of Obstetrics and Gynaecology, Faculty of Medicine, University of Medicine and Pharmacy “Gr. T. Popa”, 700115 Iasi, Romania; 7 Department of Anaesthesia and Intensive Care, University of Medicine and Pharmacy “Gr. T. Popa”, 700115 Iasi, Romania; anca.ristescu@umfiasi.ro; 8 Department of Mother and Child Medicine, University of Medicine and Pharmacy “Gr. T. Popa”, 700115 Iasi, Romania; alina.murgu@umfiasi.ro; 9 Department of Infectious Diseases, Faculty of Medicine, University of Medicine and Pharmacy “Gr. T. Popa”, 700115 Iasi, Romania; 10 St. Parascheva Clinical Hospital of Infectious Diseases, 700116 Iasi, Romania; 11 Department of Biochemistry, University of Medicine and Pharmacy “Gr. T. Popa”, 700115 Iasi, Romania; bogdan.stoica@umfiasi.ro

**Keywords:** pregnancy, urosepsis, upper UTIs, fetal distress, ureteral catheterization

## Abstract

*Background and Objectives:* Urosepsis is a significant cause of maternal and fetal mortality. While certain risk factors for urinary tract infections (UTIs) in pregnant women are well established, those associated with an elevated risk of urosepsis in pregnant women with upper UTIs remain less defined. This study aims to identify factors linked to an increased risk of urosepsis and examine urologic treatment outcomes in such cases. *Materials and Methods:* We conducted a retrospective analysis on 66 pregnant women diagnosed with urosepsis over a nine-year period. A control group included 164 pregnant women with upper UTIs, excluding urosepsis, admitted during the same timeframe. This study highlights factors potentially contributing to urosepsis risk, including comorbidities like anemia, pregnancy-related hydronephrosis or secondary to reno-ureteral lithiasis, prior UTIs, coexisting urological conditions, and urologic procedures. Outcomes of urologic treatments, hospitalization duration, obstetric transfers due to fetal distress, and complications associated with double-J catheters were analyzed. *Results:* Pregnant women with urosepsis exhibited a higher prevalence of anemia (69.7% vs. 50.0%, *p* = 0.006), 2nd–3rd grade hydronephrosis (81.8% vs. 52.8%, *p* = 0.001), and fever over 38 °C (89.4% vs. 42.1%, *p* = 0.001). They also had a more intense inflammatory syndrome (leukocyte count 18,191 ± 6414 vs. 14,350 ± 3860/mmc, *p* = 0.001, and C-reactive protein (CRP) 142.70 ± 83.50 vs. 72.76 ± 66.37 mg/dL, *p* = 0.001) and higher creatinine levels (0.77 ± 0.81 vs. 0.59 ± 0.22, *p* = 0.017). On multivariate analysis, factors associated with increased risk for urosepsis were anemia (Odds Ratio (OR) 2.622, 95% CI 1.220–5.634), 2nd–3rd grade hydronephrosis (OR 6.581, 95% CI 2.802–15.460), and fever over 38 °C (OR 11.612, 95% CI 4.804–28.07). Regarding outcomes, the urosepsis group had a higher rate of urological maneuvers (87.9% vs. 36%, *p* = 0.001), a higher rate of obstetric transfers due to fetal distress (22.7% vs. 1.2%, *p* = 0.001), and migration of double-J catheters (6.1% vs. 0.6%, *p* = 0.016), but no maternal fatality was encountered. However, they experienced the same rate of total complications related to double-J catheters (19.69% vs. 12.80%, *p* > 0.05). The pregnant women in both groups had the infection more frequently on the right kidney, were in the second trimester and were nulliparous. *Conclusions:* Pregnant women at increased risk for urosepsis include those with anemia, hydronephrosis due to gestational, or reno-ureteral lithiasis, and fever over 38 °C. While the prognosis for pregnant women with urosepsis is generally favorable, urological intervention may not prevent a higher incidence of fetal distress and the need for obstetric transfers compared to pregnant women with uncomplicated upper UTIs.

## 1. Introduction

Sepsis is a major cause of morbidity and mortality during pregnancy, both for the pregnant woman and the fetus [1]. Within the existing literature, the majority of studies have taken a broad approach, encompassing all causes of sepsis in pregnant women [2,3,4,5], and very few have specifically examined the impact of urinary tract infections (UTIs), particularly febrile infections, on the development of sepsis and its consequences for both mother and child. Similarly, factors associated with the occurrence of sepsis have been studied regardless of the site of primary infection [2,6,7]. Studies examining pyelonephritis, with or without sepsis, commonly referred to as febrile UTIs [8], have predominantly centered on pyelonephritis as a causative factor for sepsis in pregnant women and its associated antibiotic treatment [9,10,11]. Unfortunately, these studies have often failed to acknowledge that many UTIs in pregnant women are linked to underlying urological conditions or the presence of pregnancy-related hydronephrosis. Furthermore, they have not provided a comprehensive analysis of the effects of urological treatment.

Certain factors associated with an increased risk of UTI in pregnancy, including febrile infections, such as renal pelvis dilatation due to increased progesterone secretion, a history of UTIs, diabetes, anemia, socio-economic status, multiparity, and urologic disease associated with pregnancy, are known [10,11,12,13,14]. However, their role in the development of urosepsis is not well understood. Similarly, studies on the immediate prognosis of sepsis in pregnant women and its impact on mother and child include all causes of sepsis, including obstetric and digestive causes, and do not focus specifically on sepsis caused by UTIs [15].

In addition, studies looking at the efficacy and safety of the upper urinary drainage during pregnancy (e.g., by insertion of a double-J catheter or percutaneous nephrostomy) [16,17,18,19], which is the primary urologic treatment for urosepsis along with antibiotic therapy, have primarily considered pregnancy-related hydronephrosis and renal ureteral lithiasis as indications for urologic treatment but have not examined the effects of treatment in cases of urinary tract obstruction with superinfection and urosepsis.

The aim of our study was to highlight the involvement of known risk factors for UTI in the occurrence of upper UTIs and their progression to urosepsis. We sought to profile pregnant women with upper UTIs who are at risk of developing urosepsis and evaluate the immediate impact of urologic treatments for urosepsis, such as double-J catheter insertion and percutaneous nephrostomy, on both the mother and child. As a secondary objective, we compared clinical and laboratory data, as well as the etiologic agents associated with urosepsis, in patients with febrile UTIs.

## 2. Materials and Methods

We conducted a retrospective case–control study on pregnant patients hospitalized for upper UTIs at the urology clinic of Parhon University Hospital in Iasi between 1 January 2014 and 31 December 2022. The study received approval from the Hospital Ethics Committee and was registered under number 5424 on 7 July 2023. Patients data were extracted from the hospital’s electronic records, utilizing ICD-10 coding from the International Classification of Diseases, 10th edition. All pregnant women admitted to the urology clinic were included in the study. We excluded pregnant women without UTIs and patients with lower UTIs from the study.

Initially, we identified all pregnant patients with the O09 code (supervision of high-risk pregnancy) who were hospitalized during the 9-year study period, totaling 274 pregnant women. We excluded 37 patients who had been hospitalized for reno-ureteral lithiasis or pregnancy-related hydronephrosis and did not provide a urine sample showing signs of infection (such as leukocyturia or nitrites) and/or a positive urine culture. Additionally, we excluded 7 patients diagnosed with cystitis and positive urine cultures but lacking clinical and paraclinical signs of upper UTI (febrile syndrome, lumbar pain, and leukocytosis). We defined a positive urine culture in samples with ≥10^5^ CFU/mL bacterial growth; antimicrobial susceptibility testing was performed by the disk diffusion method, using EUCAST clinical breakpoint tables for the interpretation of minimal inhibitory concentrations and zone diameters.

**Figure 1:**
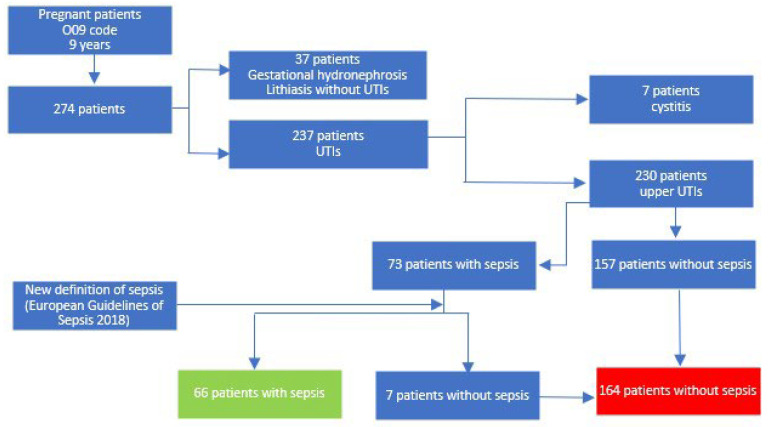
Flowchart comprising the patients’ selection process.

The remaining 230 patients with upper UTIs were divided into two groups: the study group consisted of 73 patients with urosepsis, and the control group included 157 patients with upper UTIs without urosepsis. Since our clinic adopted the new definition of sepsis, sepsis-3 [20], in 2018, we excluded 7 patients from the urosepsis group who did not meet the criteria of the new definition. Instead, we assigned them to the control group without urosepsis. We did not utilize the obstetrically modified quick Sequential Organ Failure Assessment (qSOFA) score, as per the guidelines of the Society of Obstetric Medicine Australian and New Zealand [21], as the guidelines of the European Association of Urology [22] do not mention the use of another scoring system for pregnant women. The flowchart for the selection of patients in the 2 groups is presented in Figure 1. 

From the electronic records, we analyzed the demographic characteristics of the patients upon admission. This analysis included site of the infection (on left, right kidney, or bilateral), their age, trimester of pregnancy, place of origin, parity, and comorbidities such as anemia and diabetes mellitus.

Regarding the factors associated with the risk of upper UTIs and urosepsis, we examined the presence and degree of hydronephrosis during pregnancy, secondary to gestational or reno-ureteral lithiasis, other urological conditions of the mothers, as well as the history of UTIs in pregnant women both during and outside of pregnancy, and previous endourological maneuvers during pregnancy.

To assess the grade of hydronephrosis, we utilized a four-grade ultrasound classification system developed by Onen [23]. We utilized the term mild anemia for a hemoglobin level between 9 and 11 mg/dL, the term moderate anemia for a hemoglobin level between 7 and 9 mg/dL, and severe anemia for hemoglobin under 7 mg/dL. The term ‘risk factor’ was considered in both etiological and diagnostic contexts, encompassing causal links and other personal attributes that enhance the reliability of diagnosis [24].

We also compared clinical and paraclinical characteristics between the two groups, including the degree of fever, the presence of fever exceeding 38 °C, qSOFA elements, leukocyte count, creatinine levels, C-reactive protein (CRP) levels, and the presence of septic shock upon admission among pregnant women with urosepsis. Additionally, we compared the causative pathogens responsible for UTIs in the two groups.

Concerning treatment, this study evaluated the rate of urological procedures, the insertion of a double-J catheter, percutaneous nephrostomy placement, and their localization (on left/right side, or bilateral). We performed percutaneous nephrostomy, only when we could not succeed to insert a double J catheter. 

Antibiotherapy for pregnant patients with complicated or uncomplicated UTIs was conducted according to the local protocol that followed the American College of Obstetricians and Gynecologists guidelines [25]. Blood cultures were performed for all pregnant patients with a suspicion of urosepsis according to the Royal College of Obstetricians and Gynaecologists (RCOG) guidelines [26].

We also examined the immediate treatment outcomes for both the mother and fetus, considering factors such as the incidence of fetal distress necessitating transfer to the obstetrics clinic, length of hospital stay, and the occurrence of urological complications during pregnancy in the operated pregnant women from both groups.

We defined reflux pyelonephritis as any pyelonephritis occurring in patients with a double J catheter in place, with the calcification of the double-J catheter being confirmed either by ultrasound or intraoperatively when changing the double J catheter.

All pregnant women were examined by a gynecologist during their hospitalization. In cases of fetal distress, the gynecologist decided on the patient’s transfer to the obstetrics clinic for specialized treatment. The underaged patients were also monitored by a pediatrician.

The comparison of the two groups was conducted using the Student’s *t*-test for quantitative data and the chi-square test for qualitative data. Quantitative data were described using the mean and standard deviation. The analysis of factors associated with the risk of urosepsis was performed using multivariate analyses, utilizing the SPSS version 22 software (IBM Corp., Armonk, NY, USA).

## 3. Results

During the nine years of the study, a total of 230 pregnant women with upper UTIs were admitted to the urology clinic. Out of these, 66 were pregnant with upper UTIs and urosepsis, while 164 were pregnant with upper UTIs without urosepsis. The characteristics and demographic data of the two groups are presented in Table 1.

There were no statistically significant differences between the two groups in terms of the site of the infection, mean age, trimester of pregnancy, place of origin, parity, or the incidence of diabetes. The majority of pregnant women in both groups were under 30 years of age, nulliparous, in the second trimester of pregnancy, from rural areas, and had a lower incidence of diabetes. In the study group, pregnant women had a higher rate of anemia, mainly moderate, compared with the control group. We did not encounter severe anemia in the two groups. The factors associated with the risk of urosepsis are presented in Table 2.

Patients with urosepsis had a higher incidence of hydronephrosis secondary to gestational, and a higher incidence of 2nd–3rd grade hydronephrosis for both gestational and secondary to reno-ureteral lithiasis. Also, the group with sepsis had a lower incidence of gestational 1st grade hydronephrosis compared with the non-sepsis group. Patients with urosepsis had the same rate of reno-ureteral lithiasis, the presence of other urological illnesses (one case of ureterocele in the sepsis group and one case of megaureter in non-sepsis group, both without hydronephrosis), a history of previous UTIs, and previous urological maneuvers during pregnancy. The characteristics of the two groups in terms of clinical and paraclinical data are detailed in Table 3.

The multivariate analysis of risk factors showed a statistically significant difference for 2nd–3rd grade hydronephrosis secondary to gestational and reno-ureteral lithiasis (OR 6.581, 95% CI 2.802–15.460), anemia (OR 11.612, 95% CI 4.804–28.07), and fever > 38 °C (OR 11.612, 95% CI 4.804–28.07). Patients with urosepsis had a higher fever, a higher rate of fever over 38 °C, higher qSOFA scores, higher leukocytes, C-reactive protein (CRP), and creatinine levels. Also, 12 (18.2%) of the urosepsis patients had septic shock on admission. 

Table 4 provides details about the presence of positive urine cultures, and of the types of bacteria involved, categorized by the two groups.

There was a statistically significant differences between the two groups, in favor of the urosepsis group, concerning the positivation of urine cultures.

*E. coli* was the primary pathogen responsible for the etiology of pyelonephritis in both groups, with similar incidence rates. In addition, there were no statistically significant differences in the incidence of other pathogens. Antibiotic treatment was initiated in both groups with ceftriaxone 1 g/12 h i.v.; in the case of multidrug resistance (MDR) risk (indwelling double-J catheters, hospitalization during pregnancy, treatment with antibiotics during pregnancy), treatment with meropenem 1 g/8 h i.v. was initiated; and in the case of septic shock, treatment with meropenem 1 g/12 h and vancomycin 1 g/12 h i.v. was initiated.

After the urine culture was positive, the pathogen was identified, and the antibiogram was performed, the treatment was adjusted as follows: For Gram-negative bacilli, cephalosporins were continued; for *Enterococcus* spp. and *Staphylococcus aureus*, treatment was initiated with amoxicillin + clavulanic acid 1.2 g/12 h i.v. for 7–10 days or in the case of resistance, vancomycin 1 g/12 h i.v. for 7–10 days; and for *Candida* spp., treatment was initiated with fluconazole 200 mg/12 h i.v. for 7–10 days. In the sepsis group, there were eight cases with infections with MDR pathogens (12.12%), including two with *Klebsiella* spp., five with *E. coli*, and one with *Serratia marcescens*. In the non-sepsis group, there were five (3.05%) cases with MDR infections, three with *S. aureus*, one with *Klebsiella* spp., and one case with *E. coli*. There was a statistically significant difference (*p* < 0.05) in favor of the urosepsis group regarding the frequency of MDR infections. Blood cultures were positive in 13 patients (19.7%) with urosepsis, and the results corresponded to the bacterial spectrum outlined by the urinary culture. 

In Table 5, we present patient statistics based on the surgical treatments performed.

There was a higher rate of surgical intervention in the urosepsis group, mainly due to the double-J catheter insertions, predominantly on the right side in both groups. Treatment outcomes for both groups are shown in Table 6.

The mortality rates were not included in the table due to the fact that we did not identify any fatality during the studied period.

Hospitalization was longer in the urosepsis group, with a higher rate of long hospitalization (over 7 days), and fetal disturbances requiring transfer to a maternity hospital occurred more frequently. Patients in the urosepsis group had a higher rate of migrated double-J catheters, and the development of a new urosepsis, secondary to reflux pyelonephritis. Both groups had the same rate of urological complications, such as reflux pyelonephritis, calcification of double-J catheters, septic shock during double-J catheter insertion, and failure to succeed double-J catheter insertion.

## 4. Discussion

The factors associated with an increased risk for pregnant women with upper UTIs to develop urosepsis are hydronephrosis during pregnancy or secondary to reno-ureteral lithiasis of at least 2nd grade, moderate anemia, and fever over 38 °C.

Among enteric pathogens, *E. coli* is the most commonly implicated microorganism, as indicated in other local studies [27,28,29]. Urosepsis in pregnant women increases maternal morbidity without increasing mortality compared with pregnant women with pyelonephritis without sepsis. It often requires prolonged hospitalization and admission to the intensive care unit (ICU). It can also lead to fetal distress requiring transfer to a maternity hospital.

To our knowledge, this is the first study that has analyzed the risk factors for the development of urosepsis in pregnant women with upper UTIs in a large number of patients over a significant period of time. One of the main limitations of our study is its retrospective nature, due to the small number of cases occurring each year. This required a longer study period to create a patient group suitable for statistical analysis. In addition, the urosepsis group included both patients without septic shock and those with septic shock, suggesting more severe disease. This could potentially affect treatment outcomes for both mothers and infants. However, further subdivision of the urosepsis group would have resulted in a smaller sample size, making statistical comparisons impossible. 

In both the urosepsis group and the control group, the primary source of infection was predominantly in the right kidney, because hydronephrosis during pregnancy was more pronounced on the right side than on the left [30]. Urinary stasis was identified as an important factor contributing to a high number of UTIs in both groups, whether sepsis was present or not. Furthermore, the incidence of these infections was highest in the second trimester of pregnancy, followed by the third trimester, while in the first trimester only limited cases were encountered. This pattern is consistent with the incidence and exacerbation of hydronephrosis during pregnancy [30]. The higher incidence in first-time pregnancies may be due to a lack of awareness of the need for medical screening and the importance of treating UTIs as soon as they occur.

When analyzing the differences between the two groups, we observed a higher incidence of urosepsis in patients from rural areas and in those with a higher degree of anemia. The predominance of rural residence may be explained by the lower level of education and limited access to healthcare, despite public health insurance being available in our country [31]. It is well known that pregnancy often leads to relative anemia because plasma volume grows faster than red blood cell production [32]. This anemia, especially if moderate or severe, increases the risk of sepsis [2]. Consistent with other studies, patients with urosepsis were generally younger [10], likely due to a predominantly rural background with limited access to education and healthcare. Contrary to other studies [13], we found a higher incidence in nulliparous women, possibly because nulliparous patients tend to have more severe hydronephrosis during pregnancy [30].

A significant proportion of urosepsis patients had septic shock, which may be attributed to the increased vascular risk in pregnancy due to specific physiological and immunological changes [3]. Regarding comorbidities in pregnant women, we found only one case of gestational diabetes and one case of neurologic bladder dysfunction in the urosepsis group, in addition to anemia. Both conditions are known risk factors for UTIs during pregnancy [13]. In the group without urosepsis, we observed only one case of insulin-dependent diabetes. In both groups, there were no other associated pathologies that could influence the occurrence of febrile UTIs, which is to be expected given the young age of the pregnant women.

Regarding the risk factors analyzed, in contrast to other studies that identify acute pyelonephritis without obstruction as the main cause of sepsis [10,11,13,14,33], we observed that the majority of pregnant women in both groups experienced upper urinary tract obstruction. This obstruction could be either secondary to pregnancy or to reno-ureteral lithiasis. The main difference was that pregnant women with urosepsis tended to have a higher grade of hydronephrosis than those in the group without urosepsis. During pregnancy, increased progesterone levels can lead to smooth muscle relaxation and a decrease in ureteral peristalsis [4,11]. However, when hydronephrosis occurs, it becomes a risk factor for pyelonephritis. Our study found that in patients who develop pyelonephritis, the risk of urosepsis increases if they have 2nd or 3rd grade hydronephrosis.

Pre-existing urologic conditions were present in a low percentage in both groups, and there was no significant difference in their incidence between pregnant women with urosepsis and those without urosepsis. Therefore, these diseases do not seem to influence the occurrence of urosepsis and are not considered significant risk factors for it. Also, we observed no statistically significant difference in relation to a history of UTIs and urologic procedures. This suggests that these factors do not play a significant role in the development of urosepsis in pregnant women with febrile UTIs.

Although both groups had clinical and paraclinical signs of inflammation, they were significantly increased in favor of the group with urosepsis. For both the presence of fever above 38˚C and leukocytosis, CRP and qSOFA were more elevated in the group with urosepsis. Although some studies give little importance to the presence of fever or CRP level in the detection of sepsis [21], we found a concordance between them and the presence of qSOFA, whose value in the diagnosis of sepsis in pregnant women has been proven by other studies [34]. Leukocyte count was still elevated in the urosepsis group, even if, in many pregnant women with sepsis, antibiotic treatment was started before admission. We do not have leukocytosis at the onset of sepsis, but at the moment of admission in our clinic. Instead, CRP decreases more slowly and therefore remains at high levels for a longer period of time, several days, which is an essential element in determining the presence of an inflammatory syndrome even if the leucocyte count decreased after the initiation of the antibiotic treatment. So, apart from qSOFA, we showed that in the group with urosepsis, the frequency of fever above 38 °C was higher, as were leukocytes and CRP levels. It is possible that patients with upper UTIs who develop a more severe inflammatory response are at risk for developing urosepsis. Further studies focusing on this issue will be necessary to demonstrate this. It should be mentioned that our clinic serves the entire eastern region of the country, and sometimes patients are brought by ambulance or helicopter from 200 km away, so the initiation of antibiotic treatment for sepsis begins at the hospitals where patients originally presented. We note a relatively high incidence of septic shock at admission in the urosepsis group, suggesting that worsening sepsis may occur rapidly. Another paraclinical parameter that was higher among patients with urosepsis was serum creatinine, suggesting that renal impairment occurs frequently in septic pregnant patients, even if it is not raised over 1 mg/dL, thus defining it as renal dysfunction. This indicates the necessity of rapid compensation of plasma volume, and the close monitorization of diuresis.

The main microorganisms detected in urine cultures were *E. coli*, in a similar percentage as in the reports of other studies in pregnancy [13,14,21,33], followed by *Proteus* spp., *Klebsiella* spp., *Enterococcus* spp., *Serratia* spp., germs of the gut microbiota, *S. aureus*, and *Candida* spp. Regarding MDR infections, we found a lower percentage of them compared with other studies, which is probably explained by the lower incidence of possible risk factors (age, presence of urinary catheters, multiple hospitalizations, etc.). Although the group with sepsis had a higher statistically significant percentage of MDR UTIs compared with the non-sepsis group, this incidence is lower than in other reports [35]. The fact that we could not objectify any germs in urine culture in 25% of the cases, can also be explained by the fact that antibiotic treatment was initiated in many cases in other hospitals from which patients were transferred. However, in both groups, we had a large number of patients in whom we could detect a positive urine culture, so that the conclusions could not be altered by the fact that germs were not detected in all cases.

An important aspect of our study was the fact that the overwhelming majority of patients with urosepsis had 2nd or 3rd grade hydronephrosis, in a higher percentage than in the group without urosepsis, which required surgical treatment. In our study, we had only nine cases with pyelonephritis without obstruction that developed urosepsis, five of whom had a double-J catheter in situ inserted during pregnancy for infected hydronephrosis. Thus, in the absence of hydronephrosis, the risk of progression of pyelonephritis to sepsis was low, except for the presence of double-J catheters. In all other cases, drainage was required, namely the insertion of double-J catheter or, failing that, the insertion of a percutaneous nephrostomy. A higher percentage of urologic drainage interventions was required in the group with urosepsis, due to the higher frequency of hydronephrosis with a higher grade compared to the group of pregnant women without urosepsis.

Unlike other types of sepsis in pregnant women, for which a mortality rate of 7.7% has been reported [36], our study showed no mortality and low morbidity with effective treatment. We found no maternal deaths in either group. However, despite the efficacy and safety of urologic maneuvers [37], complications and increased incidence of fetal distress and the need for transfer to a maternity hospital should not be neglected. In contrast, pregnant women without urosepsis did not experience fetal distress requiring transfer [14]. Displacement of double-J catheters, their calcification, and especially reflux pyelonephritis can be serious complications that increase maternal morbidity and the risk of fetal distress. Despite the potential complications of indwelling double-J catheter during pregnancy, the maneuver has proven necessary and has contributed decisively to the favorable evolution of pregnant women with urosepsis, becoming the mainstay of treatment, along with antibiotic therapy. The very good prognosis of patients with urosepsis can be explained by the fact that the patients were young, did not have many comorbidities, and surgical intervention and antibiotic treatment were initiated immediately.

Our study is important for obstetricians who supervise the pregnancy and for urologists who evaluate pregnant women with UTIs. They must actively look for pregnancy hydronephrosis or reno-ureteral lithiasis, initiate antibiotic treatment as early as possible at the first sign of cystitis [38] or febrile UTI, and, in the case of hydronephrosis, perform urinary drainage in the presence of an important inflammatory syndrome to avoid the evolution towards sepsis in these patients.

There is debate among obstetricians as to whether urine culture performed monthly during pregnancy is useful for the early detection of patients with UTIs or significant bacteriuria. We believe that this screening should be limited only to the category of high-risk patients, namely those with gestational hydronephrosis. If a renal ultrasound is also performed during the obstetric ultrasound examination at 14 weeks of gestation, which objectifies hydronephrosis, a monthly urine culture must be performed, thus reducing the unnecessary high number of urine tests during pregnancy. Further studies are needed to validate these findings.

## 5. Conclusions

The patients with upper UTIs who had 2nd and 3rd grade hydronephrosis had moderate anemia and fever over 38 °C, which are factors associated with an increased risk of developing urosepsis for pregnant women. 

Pregnant patients with urosepsis have a more severe inflammatory syndrome at admission than those with upper UTIs without urosepsis. *E. coli* is the main germ involved in the etiology of urosepsis, similar to the group of patients without urosepsis. 

The prognosis of pregnant women with urosepsis is good, but with a higher incidence of fetal distress and subsequent transfer to maternity hospital, and the need for urologic surgery.

## Figures and Tables

**Table 1:** Characteristics and demographic data of the two groups.

Infection Site				Sepsis (*n* = 66)	Non-Sepsis (*n* = 164)	*p* Values for chi^2^ Test
		Right		47 (71.2%)	124 (75.6%)	0.491
		Left		14 (21.2%)	26 (15.9%)	0.333
		Bilateral		5 (7.6%)	14 (8.5%)	0.811
Age (mean, standard deviation)				24.97 ± 6.51	25.18 ± 5.49	0.801 ^(t)^
Trimester of pregnancy (Nr, %)			I	5 (7.6%)	16 (9.8%)	0.604
			II	35 (53.0%)	88 (53.7%)	0.931
			III	26 (39.4%)	60 (36.6%)	0.691
Place of origin (Nr, %)			Rural	38 (57.6%)	90 (54.9%)	0.709
			Urban	28 (42.4%)	74 (45.1%)	0.709
Parity (Nr, %)			Nulliparas	45 (68.2%)	111 (67.7%)	0.942
			Parity ≥ 1	21 (31.8%)	53 (32.3%)	0.942
Comorbidities (Nr, %)	Anemia		Mild	36 (54.5%)	73 (44.5%)	0.169
			Moderate	10 (15.2%)	9 (5.5%)	0.016
			Total	46 (69.7%)	82 (50.0%)	0.006
	Diabetes mellitus			1 (1.5%)	1 (0.6%)	0.525

Table legend: ^(t)^—*p* value for *t*-test.

**Table 2:** Factors associated with the occurrence of sepsis in patients with febrile UTIs.

Gestational hydronephrosis (Nr, %)		Sepsis (*n* = 66)	Non-Sepsis (*n* = 164)	*p* Values for chi^2^ Test
	1st grade	8 (12.12%)	54 (32.93%)	0.001
	2nd–3rd grade	46 (69.70%)	52 (31.71%)	0.001
	Total	54 (81.82%)	106 (64.63%)	0.011
Hydronephrosis secondary to reno-ureteral lithiasis (Nr, %)	1st grade	2 (3.03%)	16 (9.76)	0.086
	2nd–3rd grade	8 (12.12%)	6 (3.65%)	0.015
	Total	10 (15.15%)	22 (13.41%)	0.730
Total hydronephrosis cases (Nr, %)	1st grade	10 (15.15%)	65 (39.63%)	0.001
	2nd–3rd grade	54 (81.82%)	86 (52.44%)	0.001
	Total	64 (96.96%)	151 (92.07%)	0.113
Presence of other urological disease (Nr, %)		1 (1.52%)	1 (0.61%)	0.504
History of UTIs (Nr, %)		13 (19.70%)	22 (13.41%)	0.231
History of endourologic maneuvers during pregnancy (Nr, %)		7 (10.6%)	7 (4.3%)	0.070

Table legend: UTIs—urinary tract infections.

**Table 3:** Clinical and paraclinical data of the two groups.

Fever		Sepsis (*n* = 66)	Non-Sepsis (*n* = 164)	*p* Value for Student’s *t*-test
	Mean, ±SD	38.60 ± 0.64	37.74 ± 0.69	0.001
	T > 38 °C	59 (89.4%)	69 (42.1%)	0.001 ^(^*^)^
qSOFA	Mental	57 (86.4%)	11 (6.7%)	0.001 ^(^*^)^
	Respiratory	62 (93.3%)	18 (11.0%)	0.001 ^(^*^)^
	BP < 90 mmHg	23 (34.8%)	5 (3.0%)	0.001 ^(^*^)^
Leukocytosis (mean, ±SD)		18,191 ± 6414	14,350 ± 3860	0.001
CRP (mean, ±SD)		142.70 ± 83.50	72.76 ± 66.37	0.001
Creatinine (mean, ±SD)		0.77 ± 0.81	0.59 ± 0.22	0.012
Septic shock on admission		12 (18.2%)	0 (0%)	**-**

Table legend: SD—standard deviation; CRP—C reactive protein; qSOFA—quick Sequential Organ Failure Assessment; T—temperature; BP—blood pressure. (*) *p* values for chi^2^ test.

**Table 4:** Germs involved in the etiology of UTIs in the two groups.

	Sepsis (*n* = 66)	Non-Sepsis (*n* = 164)	*p* Values for chi^2^ Test
Positive urine cultures	51 (72.3%)	103 (62.8%)	0.023
*E. coli*	36 (54.5%)	77 (47.0%)	0.297
*Klebsiella* spp.	9 (13.6%)	10 (6.1%)	0.072
*Proteus mirabilis*	1 (1.5%)	3 (1.8%)	0.867
*Pseudomonas aeruginosa*	0 (0.0%)	2 (1.2%)	0.244
*Serratia marcescens*	1 (1.5%)	0 (0.0%)	0.113
*Staphylococcus aureus*	1 (1.5%)	0 (0.0%)	0.361
*Candida* spp.	0 (0.0%)	2 (1.2%)	0.244
*Enterococcus* spp.	3 (4.5%)	9 (5.5%)	0.768

**Table 5:** Urologic maneuvers in the two groups.

		Sepsis (*n* = 66)	Non-Sepsis (*n* = 164)	*p* Values for chi^2^ Test
Double-J insertion	Right	36 (54.5%)	36 (22.0%)	0.001
	Left	11 (16.7%)	9 (5.5%)	0.010
	Bilateral	10 (15.2%)	11 (6.7%)	0.043
	Total	57 (86.4%)	56 (34.1%)	0.001
Percutaneous nephrostomy	Right	1 (1.5%)	3 (1.8%)	0.867
	Left	0 (0.0%)	0 (0.0%)	-
	Bilateral	0 (0.0%)	0 (0.0%)	-
	Total	1 (1.5%)	3 (1.8%)	0.867
Total urologic maneuvers		58 (87.9%)	59 (36.0%)	0.001

**Table 6:** Complications of the urologic treatment.

		Sepsis (*n* = 66)	Non-Sepsis (*n* = 164)	*p* Values for chi^2^ Test
Days of hospitalization	Total (mean, SD)	5.82 ± 1.69	4.48 ± 1.46	0.001 ^(t)^
	>7 days	10 (15.2%)	4 (2.4%)	0.001
Fetal distress (transfer to maternity hospital)		15 (22.7%)	2 (1.2%)	0.001
Days of hospitalization in the ICU	Nr. of patients (%)	23 (34.85%)	5 (3.05%)	0.001
	Nr. of days (mean) on patients in the ICU	2.52 ± 1	1 ± 1	0.748 ^(t)^
Surgical complications	Double-J catheter misplacement	4 (6.1%)	1 (0.6%)	0.016
	Reflux pyelonephritis	5 (7.6%)	6 (3.7%)	0.227
	Calcification of the double-J catheter after 8 weeks	2 (3.0%)	10 (6.1%)	0.320
	Septic shock after or during the urologic maneuver	1 (1.5%)	2 (1.2%)	0.860
	Failed ureteral catheterization	1 (1.5%)	2 (1.2%)	0.864
Sepsis after discharge with indwelling double-J catheter		2 (3.0%)	0 (0.0%)	0.025

Table legend: SD—standard deviation; ICU—intensive care unit; ^(t)^—*p* value for *t*-test.

## Data Availability

The data presented in this study are available on request from the corresponding author. The data are not publicly available due to local policies.
